# Vitamin D concentrations and disease activity in Moroccan children with juvenile idiopathic arthritis

**DOI:** 10.1186/1471-2474-15-115

**Published:** 2014-04-01

**Authors:** Ilham Bouaddi, Samira Rostom, Dalal El Badri, Asmae Hassani, Bouchra Chkirate, Redoine Abouqal, Bouchra Amine, Najia Hajjaj-Hassouni

**Affiliations:** 1 Department of Rheumatology, El Ayachi Hospital, University Hospital of Rabat-Salé, 11000 Salé, Morocco; 2 Department of Pediatrics, Children Hospital, University Hospital of Rabat-Salé, 10000 Rabat, Morocco; 3 Laboratory of Biostatistical, Clinical and Epidemiological Research, University Hospital of Rabat-Salé, 10000 Rabat, Morocco

**Keywords:** Juvenile idiopathic arthritis, Children, 25-hydroxyvitamin D, Disease activity

## Abstract

**Background:**

In addition to its important metabolic activities, vitamin D also contributes to the regulation of the immune system. The aim of this study was to assess the relationship between hypovitaminosis D and disease activity in Moroccan children with juvenile idiopathic arthritis (JIA).

**Methods:**

In this cross-sectional study, forty children with JIA were included, all having been diagnosed according to the classification criteria of International League of Associations for Rheumatology (ILAR). The children underwent anthropometric assessment and clinical evaluation. Disease activity was measured using the Disease Activity Score in 28 joints (DAS28) for polyarticular and oligoarticular JIA and the Bath Ankylosing Spondylitis Disease Activity Index (BASDAI) for enthesitis-related arthritis. Serum 25-hydroxyvitamin [25(OH)D] D_2_ and D_3_ were measured using radioimmunoassay (RIA). Hypovitaminosis D was defined as serum 25(OH)D <30 ng/ml.

**Results:**

The average age of participants was 11 years ± 4.23. Hypovitaminosis D was observed in 75% of patients. In univariate analyses, 25(OH)D levels were negatively associated with DAS28 for polyarticular and oligoarticular JIA. No significant relationship was found between 25(OH)D levels and BASDAI for juvenile spondylarthropathy. In multivariate linear regression analysis, no association persisted between 25(OH)D levels and DAS28.

**Conclusions:**

Our study suggested that serum levels of vitamin D were low in Moroccan children with JIA disease. Future studies with a larger population are needed to confirm our results.

## Background

In recent years, vitamin D has attracted a significant amount of attention from researchers [1]. It is estimated that as many as one billion people worldwide suffer from vitamin D deficiency or insufficiency, and this was shown to be prevalent across all age groups, genders, and geographic regions [2-4]. In addition to its important metabolic activities, vitamin D also contributes to the regulation of the immune system [5]. It has been suggested recently that vitamin D is an environmental factor that, by modulating the immune system, affects the prevalence of autoimmune diseases [6]. The immune modulatory effects of this vitamin have been subject to extensive examination, leading to recent speculation that it may play a role in select inflammatory diseases [7]. In adults, many studies have shown an inverse association between disease activity and serum levels of 25(OH)D [5]. For example, serum 25(OH)D levels were found to correlate inversely with disease activity in adults with rheumatoid arthritis (RA), in those with newly diagnosed inflammatory polyarthritis [8,9], and in ankylosing spondylitis [10]. Pelajo et al. found that 20% percent of patients who attended a pediatric rheumatology clinic were vitamin D deficient [11]. Juvenile idiopathic arthritis (JIA) is the most common chronic rheumatic disorder of childhood [12]. It is a heterogeneous and multifactorial autoimmune disease characterized by persistent joint inflammation, which manifests as swelling, pain and limitation of movement [13]. However, data is limited regarding the association between disease activity and serum levels of 25(OH)D in children with JIA. The aim of this study was to examine the association between serum levels of 25(OH)D and disease activity in Moroccan children with JIA.

## Methods

Between June and August 2011, we recruited patients diagnosed with JIA who met the classification criteria of the International League of Associations for Rheumatology (ILAR) [14]. The Departments of Rheumatology and Pediatrics of the University Hospital of Rabat-Salé carried out this cross-sectional study. Patients were excluded if they had either an additional chronic disease or were receiving treatment (except corticosteroids) that could influence vitamin D status. No patient was taking vitamin D supplementation. The Ethics Committee of university hospital center Ibn Sina approved this study and all participants’ parents provided written consent.

A detailed questionnaire was completed based on the information obtained from the patient’s medical records and by interviewing all of the participants or their parents for children under 10 years. Collected data included age, sex, subtype of JIA, disease duration, and medication (corticosteroid, non-steroidal anti-inflammatory drugs (NSAIDs), methotrexate, sulfassalazine and biological drugs). Erythrocyte sedimentation rate (ESR), C-reactive protein (CRP) and the patient’s responses to the Childhood Heath Assessment Questionnaire (CHAQ - translated and certified in Arabic) [15]. Disease activity was measured using the Disease Activity Score in 28 joints (DAS28) for polyarticular and oligoarticular JIA [16,17], and the Bath Ankylosing Spondylitis Disease Activity Index (BASDAI – translated and certified in Arabic) for juvenile spondylarthropathy [18].

### Serum 25-hydroxyvitamin D

Serum concentrations of calcium, phosphate, alkaline phosphatase, and 25-hydroxyvitamin D_2_ and D_3_ [25(OH)D] were measured. All serum samples were taken during the summer when vitamin D levels would naturally be the highest, and measurements were done in the same laboratory. Serum 25(OH)D was measured in nanograms per milliliter using radioimmunoassay. We defined levels <20 ng/ml (50 nmol/L) as vitamin D deficiency; levels from 20–29 ng/ml (50–72 nmol/L) as vitamin D insufficiency; and levels ≥30 ng/ml (75 nmol/L) as adequate [2,19].

### Statistical analysis

Analyses were performed using the software program SPSS (Windows Version 13.0, SPSS Inc., Chicago, IL). Descriptive statistics were used to assess the demographic variables and characteristics of disease activity. A linear regression was used to analyze the association between serum 25(OH)D levels and any variables that could influence serum 25(OH)D levels, including age, BMI, duration of JIA, JIA subtype, CHAQ, Patient global health, Tender joints, Swollen joints, DAS28, ESR, and BASDAI and Medications used. DAS28 and its components (except swollen joints) were included in multivariate analysis. P values ≤ 0.05 were considered significant.

## Results

Forty patients, 18 females (45%), with JIA were included in this cross sectional study. The average age of participants was 11 years ± 4.23. The median disease duration was 2 years [1-5]. The most common JIA subtypes were rheumatoid factor-positive polyarthritis (45.5%), systemic (27.5%), and oligoarticular (22.5%). Twenty three children (57.5%) were receiving corticosteroid. Twenty-two patients (55%) were using disease-modifying anti-rheumatic drugs (DMARDs). Three children (7.5%) were under biological drugs. Patients’ characteristics are presented in Table 1.

**Table 1:** Socio-demographic and clinical characteristics of the patients

Females^1^	18 (45)
Age (years)^2^	11 ± 4.23
Subtype of JIA^1^	
Systemic-onset arthritis	11 (27.5)
Oligoarthritis	9 (22.5)
Rheumatoid factor- positive polyarthritis	17 (45.5)
Rheumatoid factor- negative polyarthritis	1 (2.5)
Enthesitis-related arthritis	1 (2.5)
Psoriatic arthritis	1 (2.5)
Time since JIA onset (years)^3^	2 [1–5]
Medications used^1^	
NSAID	30 (75)
Corticosteroid	23 (57.5)
Methotrexate	16 (40)
sulfassalazine	6 (15)
Biologics	3 (7.5)
Ciclosporin	1 (2.5)
CHAQ^3^	0.5 [0–1.75]

^*1*^*Number and percentage N (%);*^*2*^*mean and standard deviation;*^*3*^*median and IQR interquartile range; NSAID: non-steroidal anti-inflammatory drugs; CHAQ: Childhood Health Assessment Questionnaire.*

**Figure 1:**
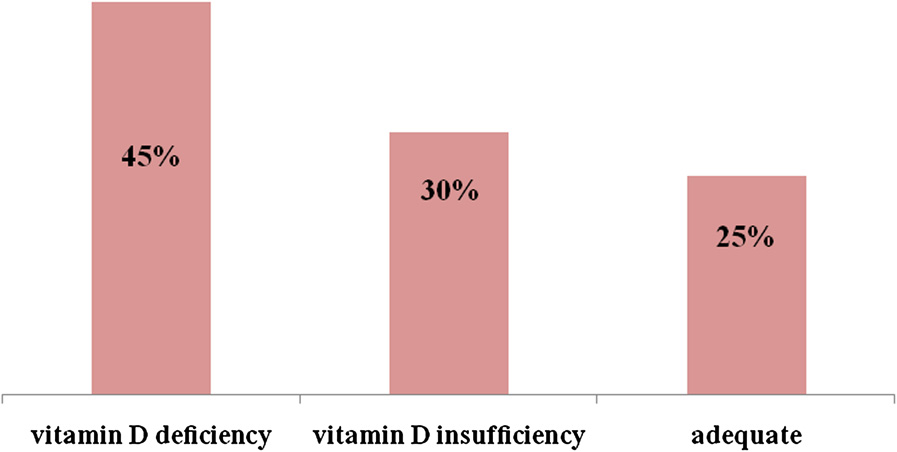
Characteristics of 25-hydroxyvitamin D.

Twenty-one patients (52.5%) were considered to have an active disease, with 48% patients having a high disease activity. The average DAS28 was 4.84 ± 1.27. Median BASDAI was 1.4 [0–2.6]. Characteristics of disease activity are presented in Table 2. 75% of children had hypovitaminosis D [25(OH)D <30 ng/ml] (Figure 1). The mean serum 25(OH)D level was 22.21 ng/ml ±10.87. Levels of serum calcium, phosphate and alkaline phosphatase were normal in all patients.

**Table 2:** Characteristics of disease activity

Tender joints^1^	3 [1–8]
Swollen joints^1^	1 [0–3]
ESR (mm/h)^1^	34.5 [25–55]
CRP (mg/l)^1^	21.5 [11–41]
Patient global assessment (cm)^1^	25 [10–40]
DAS28^2^	4.84 ± 1.27
BASDAI^1^	1.4 [0–2.6]

^*1*^*median and IQR interquartile range;*^*2*^*mean and standard deviation; DAS28: disease activity score in 28 joints; ESR: Erythrocyte sedimentation rate; CRP: C-reactive protein*; *BASDAI: Bath Ankylosing Spondylitis Disease Activity Index.*

Serum 25(OH)D levels were associated with DAS28 (p = 0.04 , β: −3.87, CI: (−7.67,-0.07) ). In the univariate linear regression analysis, serum 25(OH)D levels were associated with the following disease activity components: ESR (p = 0.05, β: −0.14, CI: (−0.28,0.004)), tender joints (p = 0.02, β: −0.79, CI (−1.47,-0.10)), and patient global health (p = 0.04, β: −0.17, CI: (−0.35,-0.004)). In multivariate linear regression analysis, no association persisted between 25(OH)D levels and DAS28, ESR, tender joints and patient global health. Number of swollen joints was not used in the multivariate analysis because it was not associated in the univariate linear regression analysis. Linear regression analysis between 25(OH)D levels and participant characteristics is presented in Table 3.

**Table 3:** Linear regression analysis between 25(OH)D levels and participant characteristics

	**Univariate analysis**			**Multivariate analysis**		
	**β**	**CI**	**p**	**β**	**CI**	**p**
Age (years)	−0. 32	(−1.15, 0.51)	0.4			
BMI	−0.15	(−0.96, 0.65)	0.7			
Time since JIA onset (years)	−0.52	(−1.74, 0.69)	0.4			
JIA subtype						
Oligoarthritis	0					
Systemic-onset arthritis	−0.92	(−8.81, 6.96)	0.8			
Polyarticular forms	−0.55	(−7.61, 6.50)	0.8			
CHAQ	−3.28	(−7.31, 0.75)	0.1			
Patient global health (cm)	−0.17	(−0.35, −0.004)	**0.04**	−0.6	(−0.50, 0.38)	0.7
Tender joints	−0.79	(−1.47, −0.10)	**0.02**	−1.23	(−3.76, 1.30)	0.3
Swollen joints	−0.67	(−1.78, 0.42)	0.2			
ESR (mm/h)	−0.14	(−0.28, 0.004)	**0.05**	−0.21	(−0.66, 0.23)	0.3
DAS28	−3.87	(−7.67, −0.07)	**0.04**	4.02	(−0.66, 0.23)	0.6
BASDAI	−1.88	(−6.40, 2.62)	0.3			
Medications used:						
NSAID	1.45	(−6.67, 9.57)	0.7			
Corticosteroid	1.08	(−6.04, 8.2)	0.7			
Methotrexate	−3.63	(−10.73, 3.46)	0.3			
Sulfassalazine	−0.04	(−9.91, 9.83)	0.9			
Biologics	1.56	(−11.81, 14.94)	0.81			

CI: confidence interval at 95%; BMI: body mass index; DAS28: disease Activity Score in 28 joints; ESR: Erythrocyte sedimentation rate; BASDAI: Bath AS Disease Activity Index; NSAID: non-steroidal anti-inflammatory drugs; CHAQ: Childhood Health Assessment Questionnaire.

## Discussion

To the best of our knowledge, this is the first study in Morocco, let alone in an Arabic and/or African country, concerning hypovitaminosis D and disease activity in children with JIA. When measured by DAS28, our results showed an association between serum 25(OH)D levels and JIA disease activity. On the other hand, no significant relationship was found between BASDAI and 25(OH)D. In multivariate linear regression, no association persisted between 25(OH)D and disease activity components. The small sample size of children; and the duration of illness may explain this result. With the average onset time being two years earlier, many of the children were taking medication that would modify the disease course. One study also examining serum 25(OH)D levels and disease activity in children and adolescents was performed by Pelajo et al., which found no association between the two variables [20]. Patel et al. examined patients who had been recently diagnosed in the early stage of inflammatory polyarthritis, 45% of whom were classified as having RA for no more than 1 year [8]. Like us, they found a strong inverse association between baseline levels of both serum 25(OH)D and disease activity, as assessed by DAS28. Two studies completed in the last five years have highlighted the association between autoimmunity and vitamin D deficiency [21,22]. Cutolo et al. reported that vitamin D is implicated in the pathogenesis of different autoimmune disease [23]. Those results support the research of numerous other studies, which found that the immune system is characterized by low serum levels of vitamin D that correlate to the severity of the disease [4,24]. In unadjusted analysis, a 2010 study found that vitamin D concentrations were inversely associated with baseline pain (p = 0.04), swollen joints (p = 0.04), and DAS28 (p = 0.05) in African American patients with early stage RA [7]. When comparing RA patients to healthy controls, 5 studies revealed lower levels of 25(OH)D in the patients [7,22,25,26], while 2 studies did not find such a distinction [27,28]. Looking beyond RA to other autoimmune disease, Becker et al. found that 34 of 57 systemic lupus erythematosus (SLE) patients who had high disease activity also had severe vitamin D depletion [29].

Our study showed a high level of hypovitaminosis D (75%) in children with JIA. In studies comparing healthy children to children with JIA, vitamin D deficiency has been noted in children with JIA [30]. Epidemiological data indicates that up to 60% of patients with RA have 25(OH)D levels <50 nmol/L, and 16% have levels would be classified as vitamin D deficiency (<12.5 nmol/L) [31]. Pelajo et al. compared two groups, one with and one without autoimmune disorders [11]. They found that of those individuals in the autoimmune disorders group, 23% had vitamin D deficiency [serum 25(OH)D < 20 ng/ml], and of those in the non-autoimmune group, 14% were vitamin D deficient. Even though there has been extensive evidence of vitamin D deficiency in association with autoimmune rheumatologic disorders in adults, little information is available relating to children [4].

Despite the methodological limitations of this study (small sample size, cross-sectional, single-center study (in El ayachi hospital, there is more recruitment of late and polyarticular forms. however, in paediatric hospital, they recruit more early and oligoarticular forms which constitu a bias of recruitment) and absence of control), our results could support the studies concerning the association between serum 25(OH)D levels and disease activity in children and adolescents with JIA.

## Conclusion

Our study showed that three-quarters of children with JIA had hypovitaminosis D. This study adds evidence to the growing knowledge regarding vitamin D and autoimmunity. Additional research, with a larger sample of children, is needed to confirm our findings.

## Competing interests

The authors declare that they have no conflict of interest.

## Authors’ contributions

This work was carried out in collaboration between all authors. All authors read and approved the final manuscript.

## Pre-publication history

The pre-publication history for this paper can be accessed here:

http://www.biomedcentral.com/1471-2474/15/115/prepub
